# Molecular origins of absorption wavelength variation among phycocyanobilin-binding proteins

**DOI:** 10.1016/j.bpj.2024.08.001

**Published:** 2024-08-08

**Authors:** Tomoyasu Noji, Keisuke Saito, Hiroshi Ishikita

**Affiliations:** 1 Department of Applied Chemistry, The University of Tokyo, Bunkyo-ku, Tokyo, Japan; 2 Research Center for Advanced Science and Technology, The University of Tokyo, Meguro-ku, Tokyo, Japan

## Abstract

Phycocyanobilin (PCB)-binding proteins, including cyanobacteriochromes and phytochromes, function as photoreceptors and exhibit a wide range of absorption maximum wavelengths. To elucidate the color-tuning mechanisms among these proteins, we investigated seven crystal structures of six PCB-binding proteins: Anacy_2551g3, AnPixJg2, phosphorylation-responsive photosensitive histidine kinase, RcaE, Sb.phyB(PG)-PCB, and Slr1393g3. Employing a quantum chemical/molecular mechanical approach combined with a polarizable continuum model, our analysis revealed that differences in absorption wavelengths among PCB-binding proteins primarily arise from variations in the shape of the PCB molecule itself, accounting for a ∼150 nm difference. Remarkably, calculated excitation energies sufficiently reproduced the absorption wavelengths of these proteins spanning ∼200 nm, including 728 nm for Anacy_2551g3. However, assuming the hypothesized lactim conformation resulted in a significant deviation from the experimentally measured absorption wavelength for Anacy_2551g3. The significantly red-shifted absorption wavelength of Anacy_2551g3 can unambiguously be explained by the significant overlap of molecular orbitals between the two pyrrole rings at both edges of the PCB chromophore without the need to hypothesize lactim formation.

## Significance

Cyanobacteriochromes and phytochromes utilize phycocyanobilin (PCB) as a light-absorbing pigment, exhibiting significant variations in absorption wavelengths spanning hundreds of nanometers. Employing a systematic approach based on protein crystal structures, we accurately determined absorption wavelengths of PCB-binding proteins and identified key factors influencing their variations. Variances in absorption wavelengths primarily originate from PCB conformation. The coplanarity of PCB reliably predicts wavelengths of PCB-binding proteins, with the exception of Anacy_2551g3. The remarkably lengthened wavelength of Anacy_2551g3 arises from molecular orbital overlap rather than hypothesized lactim formation. These findings deepen our understanding of color-tuning mechanisms, elucidating the relationship between molecular structure and light perception in biological systems.

## Introduction

Phycocyanobilin (PCB)-binding proteins, including cyanobacteriochromes and phytochromes found in various organisms such as cyanobacteria, plants, algae, bacteria, and fungi, serve as crucial photoreceptors (1,2). These proteins share a common chromophore, tetrapyrrole PCB, which consists of rings A, B, C, and D, along with two propionic groups at rings B and C, covalently attached via cysteine at the ring-A moiety to the protein environment. In many cases, the four pyrrole nitrogen sites are protonated, stabilizing a lactam form (e.g., (3,4)). The fundamental photoisomerization process of PCB-binding proteins involves the double bond between rings C and D, altering the electronic structure of the chromophore within picoseconds (e.g., (5,6)). This process typically results in reversible conversion between two distinct states: the green-absorbing (Pg) state and the red-absorbing (Pr) state, thereby serving as a photoreceptor and modulating biological activity or signaling function.

Among cyanobacteriochromes, PixJs from *Anabaena* sp. PCC 7120 (AnPixJg2) functions as a putative phototaxis regulator (3), while RcaE from *Microchaete diplosiphon* optimizes light absorption maxima of the photosynthetic antenna complex phycobilisome through chromatic acclimation (4). RcaE controls phycobilisome gene expression via phosphorylation of RcaF and RcaC under red light (7). Crystal structures in the Pr state have been reported for these cyanobacteriochromes (3,4).

In contrast, Slr1393 from *Synechocystis* sp. PCC6803 is a unique cyanobacteriochrome with both Pg and Pr structures reported (8). According to Köhle et al., the significant increase in absorption wavelength during the Pr (532 nm (8)) to Pg (649 nm (8)) transition was associated with changes in charge distribution, shifting from the localization of the positive charge on ring B in the Pr state to its delocalization over rings B and C in the Pg state (9). Theoretical studies by Wiebeler et al. suggested that twisting of ring D alters the conjugation length effectively and thereby plays a crucial role in its red/green spectral tuning in Slr1393 (10).

As a phytochrome, Sb.phyB(PG)-PCB, B-type phytochrome from *Sorghum bicolor*, acts as a principal sensory photoreceptor in light perception, while A-type phytochrome serves as an extraordinarily sensitive sensory photoreceptor for seeds and seedlings. The crystal structure in the Pr state has been reported for Sb.phyB(PG)-PCB (11). Similar features are conserved in other photosensory proteins. For phosphorylation-responsive photosensitive histidine kinase (PPHK), a multidomain sensory histidine kinase from cyanobacterium *Leptolyngbya* sp. JSC-1, the Pg-state crystal structure has been reported (12). In all these PCB-binding proteins, conversion between the Pg and Pr states is essential for light sensing. The Pg state typically exhibits absorption maximum wavelengths of 530–560 nm, while the Pr state exhibits absorption wavelengths lengthened by ∼100 nm, ranging from 640 to 670 nm.

Remarkably, Anacy_2551g3, a recently identified cyanobacteriochrome from *Anabaena cylindrica* PCC 7122, exhibits sensitivity to far-red spectrum regions, with an absorption peak at 728 nm (13), over 50 nm longer than that of other typical Pr-state PCB-binding proteins. To interpret this remarkable absorption wavelength, Bandara et al. hypothesized that in the PCB chromophore of Anacy_2551g3, the keto O site of ring A is protonated due to deprotonation of the pyrrole N site of ring B, resulting in the lactim conformation (13). This hypothesis may be supported by the observation that isolated model PCB compounds with two protonated propionic groups exhibit longer absorption wavelengths in the lactim conformations (14), although the two propionic groups are likely fully deprotonated due to salt-bridge and H-bond formations in the protein environment of PCB-binding proteins, as identified in the crystal structures.

To gain deeper insights into the color-tuning mechanisms of PCB-binding proteins, theoretical analyses can provide valuable insights. According to Wiebeler and Schapiro (15), calculations of absorption wavelengths of PCB-binding proteins using a quantum chemical/molecular mechanical (QM/MM) approach were first reported in (10) in 2019. Thus, theoretical investigations into PCB-binding proteins still remain limited to specific examples (e.g., (8,9,10)). To our knowledge, there are no examples demonstrating the application of a single consistent methodology to systematically explain differences in absorption wavelengths among various PCB-binding proteins. Therefore, it remains unclear how each key factor of the protein environment uniquely influences the resulting absorption wavelength among a series of PCB-binding proteins. These factors include electrostatic interactions with the protein environment, such as hydrogen-bond interactions with polar residues, π-stacking interactions with aromatic residues, chromophore planarity (facilitating efficient delocalization of π-electrons and affecting the absorption wavelength), and the impact of solvation loss on the chromophore in the protein matrix. To pinpoint the factors that differentiate absorption wavelengths among these seven PCB-binding proteins in a range of ∼200 nm, we investigated seven crystal structures of PCB-binding proteins based on the systematic methodology commonly used for analyzing light-absorbing proteins (e.g., microbial rhodopsins (16,17)), using a QM/MM framework combined with a polarizable continuum model (PCM).

## Materials and methods

### Atomic coordinates and partial charges

The atomic coordinates of the following PCB-binding proteins were taken from the X-ray diffraction crystal structures: Anacy_2551g3 in the far-red-absorbing (Pfr) state (from *Anabaena* sp. PCC 7122; PDB: 6UV8) (13); AnPixJg2 in the Pr state (from *Anabaena* sp. PCC 7120; PDB: 3W2Z) (3); phosphorylation-responsive photosensitive histidine kinase in the Pg state (from *Leptolyngbya* sp. JSC-1; PDB: 6OAP) (12); RcaE in the Pr state (from *Microchaete diplosiphon*; PDB: 7CKV) (4); Sb.phyB(PG)-PCB in the Pr state (from *Sorghum bicolor*; PDB: 6TBY) (11); and Slr1393g3 in the Pg (from *Synechocystis* sp. PCC6803; PDB: 5M82) and Pr (PDB: 5DFX) states (8). Hydrogen atoms were generated and energetically optimized using CHARMM (18). Atomic partial charges of amino acids were adopted from the all-atom CHARMM22 (19) parameter set. The atomic charges of PCB were determined by fitting the electrostatic potential in the neighborhood of this molecule using the restrained electrostatic potential (RESP) procedure (20) (Table S1). The four nitrogen sites of PCB were considered to be protonated based on analysis of nuclear magnetic resonance (NMR) spectroscopy of RcaE (4) and AnPixJ (3).

### Protonation pattern

The protonation pattern of the titratable residues was determined by solving the linear Poisson-Boltzmann equation using the MEAD program (21). All calculations were conducted at 300 K, pH 7.0, and an ionic strength of 100 mM, with dielectric constants of 4 for the protein interior and 80 for bulk water. To calculate the p*K*_a_ values of titratable sites in the protein, the calculated p*K*_a_ difference between the protein site and the reference system was added to the known reference p*K*_a_ value (e.g., 4.0 for Asp (22)). The experimentally measured p*K*_a_ values used as references were 12.0 for Arg, 4.0 for Asp, 9.5 for Cys, 4.4 for Glu, 10.4 for Lys, 9.6 for Tyr (22), 7.0 and 6.6 for the N_ε_ and N_δ_ atoms of His, respectively (23,24,25), and 4.8 for the propionic group (26) of PCB. During titration, all other titratable sites were fully equilibrated to the protonation state of the target site. Protonation patterns were sampled using a Monte Carlo method with Karlsberg (27). The linear Poisson-Boltzmann equation was solved through a three-step grid-focusing procedure at resolutions of 2.5, 1.0, and 0.3 Å. Monte Carlo sampling provided the probabilities ([protonated] and [deprotonated]) for the two protonation states.

The resulting protonation states for titratable residues in the PCB-binding proteins were their standard protonation states, i.e., protonated basic and deprotonated acidic residues, except for those listed in Table S2. Histidine residues that were doubly protonated are also listed in Table S2. In addition, the resulting protonation states were essentially consistent with those calculated using PROPKA 3 (28,29), with one or two residues exhibiting discrepancies in each protein (Table S2). Most of these residues are more than 10 Å away from PCB, and their influences on the calculated absorption wavelength are likely marginal. However, three residues, His119 in the AnPixJg2 structure (protonated in the present approach and deprotonated in PROPKA 3), His529 in the Pr-state Slr1393g3 structure (protonated in the present approach and deprotonated in PROPKA 3), and Glu143 in the Pr-state RcaE structure (deprotonated in the present approach and protonated in PROPKA 3), are close to PCB, and the discrepancy in the protonation state is likely crucial (Fig. S1).

1) In the AnPixJg2 structure, His119 forms an H-bond with the deprotonated propionic group of PCB (2.9 Å; Fig. S1 *b*). Thus, protonated His119, as calculated in the present approach, appears more reasonable than deprotonated His119 predicted by PROPKA 3.
2) This also holds true for His529 in the Pr-state Slr1393g3 structure, which forms an H-bond with the deprotonated propionic group of PCB (2.7 Å; Fig. S1 *g*).
3) In the Pr-state RcaE structure, Glu143 is adjacent to the pyrrole N of PCB (2.7 Å; Fig. S1 *d*). In RcaE, the four pyrrole nitrogen sites are protonated (4). However, PROPKA 3 attempts to titrate pyrrole N sites and results in deprotonated pyrrole N sites. Consequently, PROPKA 3 predicted Glu143 to be protonated, whereas the present approach resulted in deprotonated Glu143 in the presence of protonated pyrrole N sites (Table S2).

Based on these observations, the following calculations were based on the protonation states determined using the present approach rather than those predicted by PROPKA 3.

### QM/MM calculations

For geometry optimization, QM/MM calculations were performed to investigate the absorption energy of PCB-binding proteins. The restricted density functional theory (DFT) method, using the B3LYP functional and LACVP^∗^ basis sets with the QSite program (30), was employed to obtain the QM/MM-optimized geometry. The QM region was defined to include PCB with two cysteine side chains and residues involved in the H-bond network of PCB (Table S3). All atomic coordinates were fully relaxed in the QM region. In the MM region, optimization of H atom positions was performed using the OPLS2005 force field (31), while heavy atom positions were held fixed. Detailed atomic coordinates of the QM/MM-optimized geometry are provided in the supporting material.

Using the QM/MM-optimized geometry, the absorption energy was calculated employing the PCM method. In this method, polarization points were positioned on spheres with a radius of 3.0 Å from the center of each atom to account for possible water molecules in the cavity. A dielectric constant of 78 was applied for the bulk water region, explicitly considering electrostatic and steric effects created by the protein environment. The restricted time-dependent DFT (TDDFT) was applied using the CAM-B3LYP functional (32) and 6-31G^∗^ basis sets, with a range-separation parameter *μ* of 0.33 (32), *α* of 0.19, and *β* of 0.46 (i.e., polarizable TDDFT-QM/MM/PCM).

For further analysis of the electrostatic influence of each residue on the absorption energy, a smaller QM region was used to analyze the electrostatic contributions of residues involved in the H-bond network to the absorption wavelength. Consequently, the QM region was reduced to include only PCB with cysteine. To calculate the influence of each residue on the absorption energy of the QM region, the absorption energy was calculated in the absence of the atomic charges of the focusing residue, and the difference from the original absorption energy was determined as the electrostatic contribution of the residue to the absorption energy.

## Results and discussion

### Overview of absorption wavelengths of PCB-binding proteins

The protonation pattern of the titratable residues, calculated by solving the linear Poisson-Boltzmann equation, consistently indicates deprotonation of the two propionic groups of PCB in all PCB-binding proteins (Table S4). This deprotonation occurs due to the formation of H-bonds, including salt bridges, with the protein environments. Therefore, in the present study, propionic groups are uniformly treated as deprotonated in each protein environment of the PCB-binding proteins.

**Figure 1:**
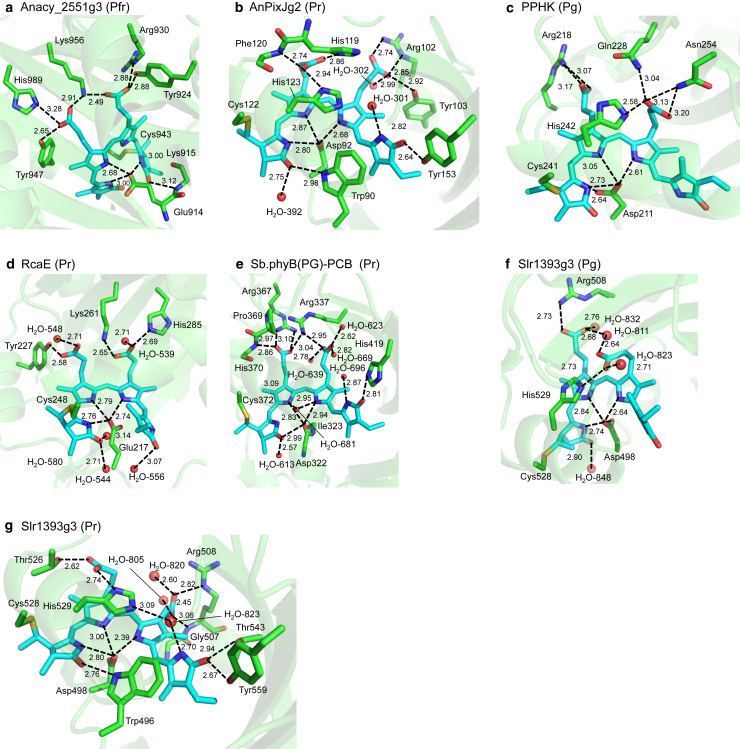
PCB chromophores in the QM/MM-optimized structures of the PCB-binding proteins. PCB and H-bond network groups, considered quantum-chemically in QM/MM/PCM calculations (i.e., QM region), are shown explicitly. Dotted lines indicate H-bonds. (*a*) Anacy_2551g3 in the Pfr state. (*b*) AnPixJg2 in the Pr state. (*c*) PPHK in the Pg state. (*d*) RcaE in the Pr state. (*e*) Sb.phyB(PG)–PCB in the Pr state. (*f*) Slr1393g3 in the Pg state. (*g*) Slr1393g3 in the Pr state.

**Figure 2:**
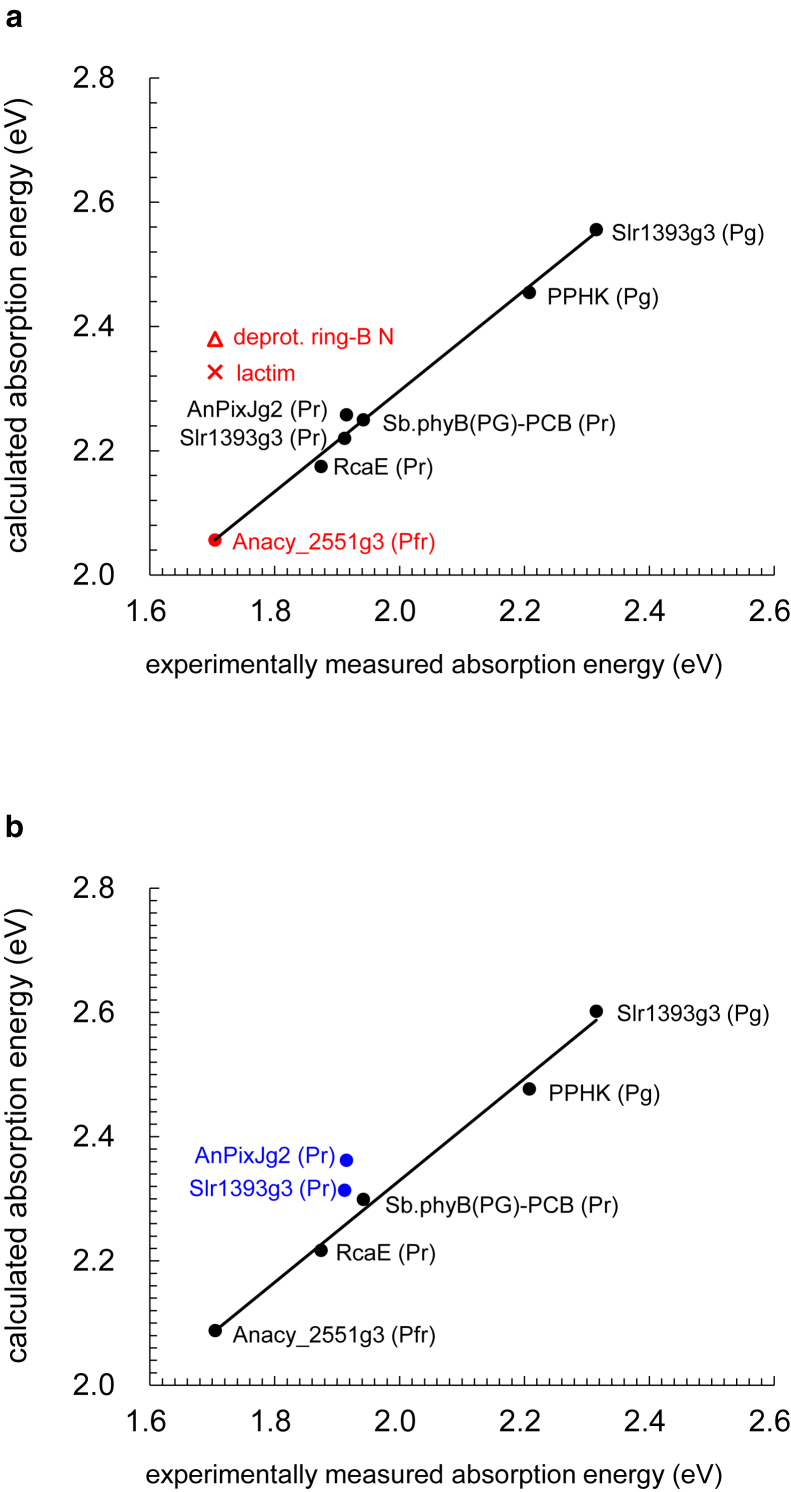
Comparison of calculated absorption energies and experimentally measured absorption energies for PCB-binding proteins. (*a*) Absorption energies calculated considering the residues involved in the H-bond network of the PCB moiety quantum-chemically (i.e., QM region). Anacy_2551g3 is highlighted in red. The symbol × represents Anacy_2551g3, calculated assuming a lactim conformation as hypothesized by Bandra et al. (lactim 3 in (13); see below). Note that the ring-B N site is deprotonated in this lactim conformation. The open triangle represents Anacy_2551g3 with the deprotonated ring-B N site. (*b*) Absorption energies calculated considering the residues involved in the H-bond network of the PCB moiety electrostatically (i.e., MM region). The Pr-state structures of AnPixJg2 and Slr1393g3, with tryptophan adjacent to the PCB chromophore, are highlighted in blue for clarity. Note that these results remained unchanged when geometry optimization was perfumed using the CAM-B3LYP functional and LACVP^∗^ basis sets with a range-separation parameter *μ* of 0.33, *α* of 0.19, and *β* of 0.46 (Fig. S2).

In the present QM/MM/PCM calculation, when considering all residues involved in the H-bond network of the PCB moiety quantum-chemically (i.e., QM region, Fig. 1), the resulting lowest excitation energies (*E*_TDDFT_) and the experimentally measured absorption energies (*E*_expl_) exhibit a significant correlation (coefficient of determination *R*^2^ = 0.99). This correlation is best described by the following equation (Fig. 2 *a*):

(1) $$E_{\mathrm{expl}}\left(\mathrm{eV}\right)=1.235E_{\mathrm{TDDFT}}-0.836\text{.}$$

Thus, following the methodology outlined here, one can effectively reproduce the experimentally measured absorption wavelengths of the PCB-binding proteins by calculating the lowest excitation energies and applying Eq. 1 (Table 1). Note that the overall correlation between *E*_TDDFT_ and *E*_expl_ essentially remains unchanged even when calculated without the PCM (coefficient of determination *R*^2^ = 0.94, Fig. S3). The slight decrease in correlation without the PCM suggests that variations in PCB absorption wavelengths are predominantly influenced by factors other than the solvation accessible surface of PCB (see PCB conformation below).

**Table 1:** Calc. and Expl. absorption wavelengths of PCB in the presence (protein) or absence (water) of the protein environment (nm)

	State	Expl.	Calc.		Contributions		
			Protein	Water	Protein environment	Residue charge	Loss of solvation
Anacy_2551g3	Pfr	728^a^	728	683	45	60	−15
AnPixJg2	Pr	648^b^	635	608	27	59	−32
PPHK	Pg	562^c^	565	574	−9	46	−55
RcaE	Pr	662^d^	670	636	34	45	−11
Sb.phyB(PG)-PCB	Pr	639^e^	638	635	3	60	−57
Slr1393g3	Pg	532^f^	534	533	2	42	−40
Slr1393g3	Pr	649^f^	651	613	38	59	−21

Contributions from protein environments, including atomic charges of the protein side chain and backbone, and loss of solvation for PCB to the absorption wavelengths are also summarized (nm). Absorption energy values are listed in Table S5. Calc., calculated; Expl., experimentally measured; PPHK, phosphorylation-responsive photosensitive histidine kinase.

a Bandara et al. (13).

b Tachibana et al. (33).

c Shin et al. (12).

d Okuda et al. (34).

e Nagano et al. (11).

f Xu et al. (8).

QM/MM/PCM calculations reveal that the lowest excitation energies are mainly determined by the energy gap between the highest occupied molecular orbital (HOMO) and the lowest unoccupied molecular orbital (LUMO). This observation is supported by a high correlation between the calculated energy differences of HOMO-LUMO and the experimentally measured absorption energies, with a coefficient of determination *R*^2^ = 0.92 (Fig. S4). This trend is consistent with observations for chromophores in microbial rhodopsins (16, 35) and photoactive yellow protein (36).

**Figure 3:**
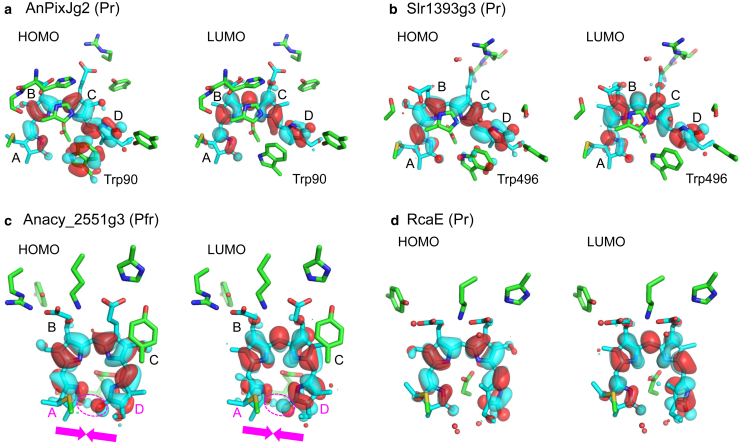
Distributions of HOMO and LUMO over PCB chromophores. (*a*) Pr-state structure of AnPixJg2. (*b*) Pr-state structure of Slr1393g3. (*c*) Pfr-state structure of Anacy_2551g3. (*d*) Pr-state structure of RcaE. Pink arrows and ovals indicate overlap of molecular orbitals between rings A and D. For distributions in other PCB-binding proteins, see Fig. S5.

The delocalization of HOMO and LUMO over the PCB moiety is observed in all PCB-binding proteins (Fig. S5). Remarkably, when tryptophan is positioned adjacent to PCB (e.g., Trp90 in the Pr-state AnPixJg2 structure and Trp496 in the Slr1393g3 structure), the HOMO extends over the tryptophan moiety as well (Fig. 3). This result is evident from the parallel orientation of the tryptophan plane to the ring-D plane of PCB in the Pr-state AnPixJg2 and Slr1393g3 structures, forming a π-stacking conformation (3,33) (Figs. 1 and 3). Thus, the HOMO energy level of tryptophan matches that of PCB, facilitating hybridization of their HOMOs. Consequently, this hybridization results in an elevation of the HOMO energy level of the PCB-tryptophan chromophore, thereby contributing to the lengthening of the absorption wavelength in the Pr-state structures of AnPixJg2 and Slr1393g3. When the tryptophan is not considered quantum-chemically (i.e., excluded from the QM region) but is instead considered electrostatically (i.e., MM region), the calculated absorption energies for these two Pr-state structures of AnPixJg2 and Slr1393g3 become elevated (i.e., blue-shifted absorption wavelengths compared to the experimentally measured absorption wavelengths) (Fig. 2 *b*). Thus, the presence of the adjacent tryptophan contributes to the increase in the absorption wavelength.

These findings suggest that tryptophan plays a crucial role as a prerequisite component of the PCB chromophore to appropriately achieve the experimentally measured absorption wavelength of PCB-binding proteins. However, it is important to note that the hybridized HOMO between PCB and tryptophan is not the sole determinant for the longer absorption wavelength in the Pr state compared to the Pg state for Slr1393g3 (see also below). As suggested by Köhle et al., the significant increase in absorption wavelength during the Pr-to-Pg transition was likely associated with more delocalized charge distribution over rings B and C in the Pg state (9).

### Key factors contributing to variations in absorption wavelengths among PCB-binding proteins

Below, we focus on the following three factors contributing to variations in absorption wavelengths among PCB-binding proteins: 1) PCB conformation, which affects the conjugation length and is determined by interactions with the protein environment; 2) loss of solvation for PCB in the protein environment, which leads to destabilization of the charged or polarized state of PCB; and 3) electrostatic interactions between PCB and protein residues.

#### PCB conformation

The calculated absorption wavelength of PCB in PCB-binding proteins shows a strong correlation with the absorption wavelength of isolated PCB molecules in water, i.e., in the absence of the protein environment (coefficient of determination *R*^2^ = 0.95; Fig. S6). This indicates that the shape, or conformation, of PCB is a primary determinant of the variation in absorption wavelengths among PCB-binding proteins. The significance in the conformation of PCB is evident by the observation that the PCB conformation in the Pg state of Slr1393g3 already exhibits the shortest wavelength (533 nm) in the absence of the protein environment, while the PCB conformation in Anacy_2551g3 already exhibits the longest wavelength (683 nm) in the absence of the protein environment (Table 1).

**Figure 4:**
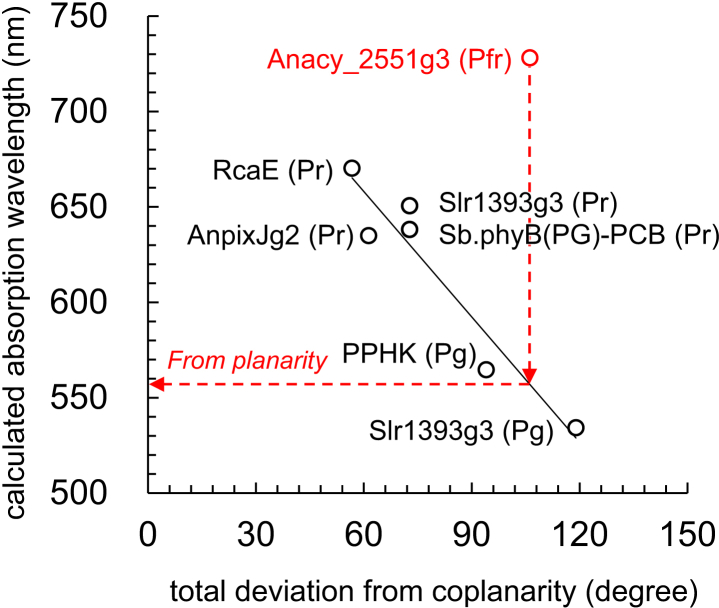
Comparison of calculated absorption wavelength and planarity of PCB. Absorption energies are calculated considering the residues involved in the H-bond network of the PCB moiety quantum-chemically (i.e., QM region). The horizontal axis, total deviation from coplanarity, indicates the sum of differences in torsion angles between rings A and B, B and C, and C and D in the QM/MM-optimized structures. These torsion angles were calculated as the angle between the normal vectors of two ring planes. The normal vector for each ring was determined by three atoms: (C1C, NC, C4C) for ring A, (C1D, ND, C4D) for ring B, (C1A, NA, C4A) for ring C, and (C1B, NB, C4B) for ring D. Anacy_2551g3 is highlighted in red. Red dotted arrows indicate the absorption energy estimated from the planarity of PCB in Anacy_2551g3. See Fig. S7 for PCB conformations in QM/MM-optimized structures of the PCB-binding proteins.

In particular, the planarity of PCB has been suggested to be a crucial factor influencing the absorption wavelengths in PCB-binding proteins (13). The planar structure of PCB facilitates efficient delocalization of π-electrons over the tetrapyrrole ring system, leading to longer absorption wavelengths compared to nonplanar conformations. This characteristic is also evident in the present QM/MM calculation, where the sum of differences in torsion angles between rings A and B, B and C, and C and D (i.e., “total deviation from coplanarity” (8,37)) exhibits a high correlation of the absorption wavelength in the PCB-binding protein when excluding Anacy_2551g3 (discussed below; coefficient of determination *R*^2^ = 0.91; Fig. 4).

Although these findings might initially imply that the interaction between PCB and the protein environment is not critical, it is important to recognize that each PCB conformation is influenced by interactions with the specific protein environment of the PCB-binding protein. These interactions, including electrostatic interactions (e.g., H-bonds) and van der Waals contacts, ultimately shape the PCB conformation, thus impacting its absorption wavelength.

#### Loss of solvation for PCB in the protein environment

The loss of solvation for PCB in the protein environment weakens its polarization, thereby affecting its excitation energy. QM/MM/PCM calculations indicate that this influence is more pronounced on the absorption wavelength compared to the influence of electrostatic interactions with the protein environment, resulting in the differentiation of absorption wavelengths by approximately 50 nm among the PCB-binding proteins (Table 1).

**Figure 5:**
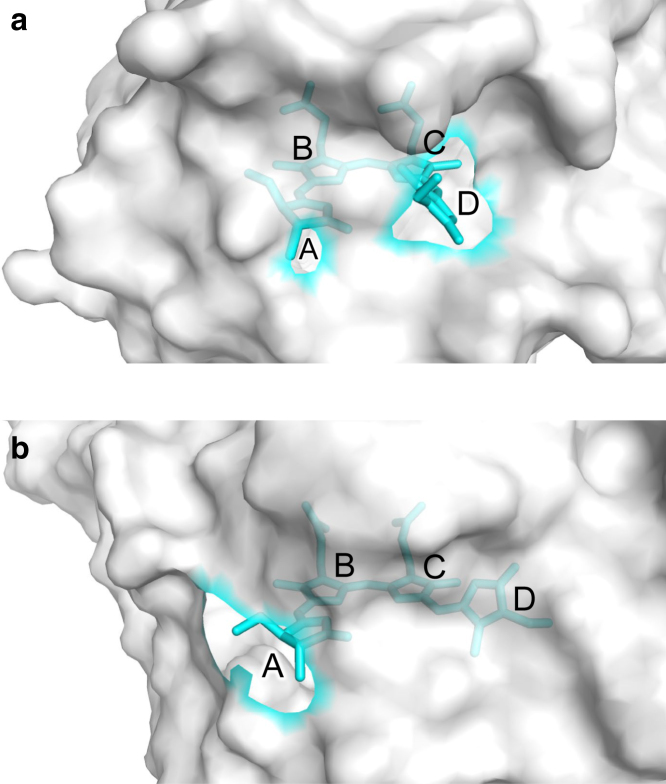
Protein bulk surface at the PCB binding moiety. (*a*) RcaE. (*b*) Sb.phyB(PG)-PCB. The protein surfaces are represented by spheres. The region outlined in cyan on the protein surface indicates the crevice where PCB is exposed.

Notably, the impact of solvation loss is smallest in RcaE and Anacy_2551g3 (11–15 nm shortening) and largest in Sb.phyB(PG)-PCB (57 nm shortening) (Table 1). The variation in solvation loss impact on absorption wavelength can be explained by differences in the exposure of the PCB chromophore, especially the ring-D moiety. The burial of the ring-D moiety in the protein environment shields its connecting ring-C and ring-B moieties from bulk water. For instance, in RcaE, the ring-D moiety is largely exposed to the protein bulk surface, resulting in minimal loss of solvation in the protein environment (Fig. 5 *a*). Conversely, in Sb.phyB(PG)-PCB, the ring-D moiety is fully shielded by the protein environment, leading to a significant loss of solvation of the PCB chromophore (Fig. 5 *b*). As the PCB molecule adopts a more cyclic, ring-like shape, rings A and D are more likely to be exposed together to the protein bulk surface, as observed in RcaE and Anacy_2551g3. Thus, the degree of loss of solvation for PCB is directly linked to the importance of the shape of isolated PCB molecules in determining the absorption wavelength (e.g., Fig. S6), cooperatively influencing the shift in absorption wavelength in the protein environment.

#### Electrostatic interactions with residues

**Figure 6:**
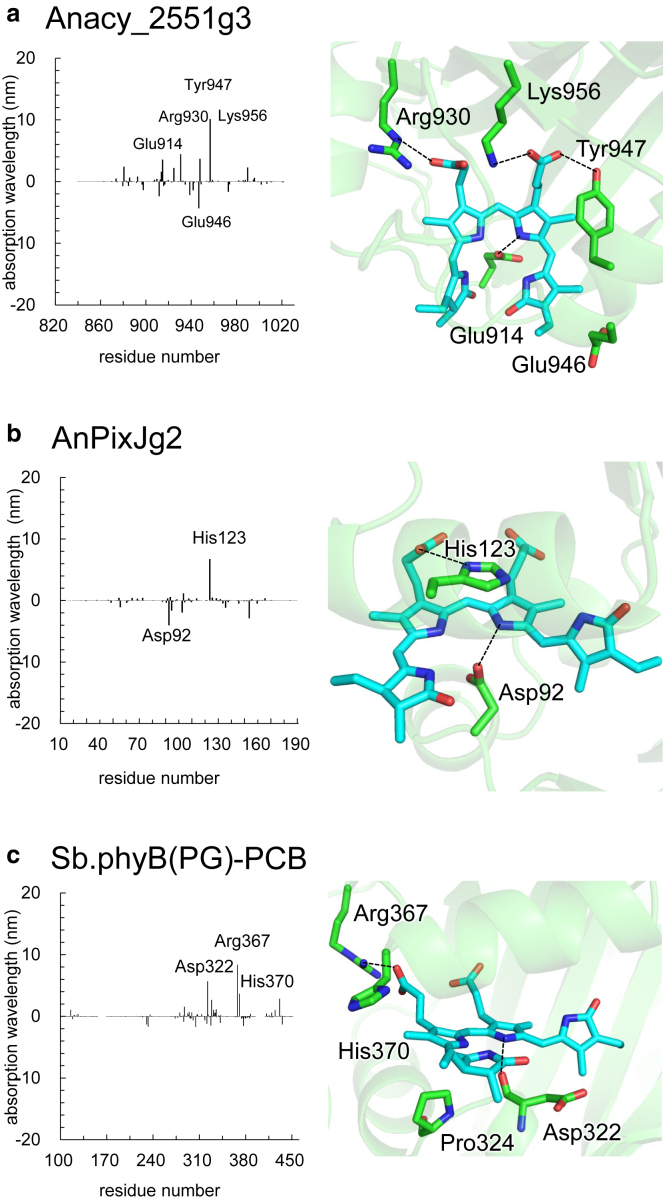
Contributions of residues to the absorption wavelength (nm). Protein bulk surface at the PCB binding moiety. (*a*) Anacy_2551g3. (*b*) AnPixJg2. (*c*) Sb.phyB(PG)-PCB. For contributions of residues in other PCB-binding proteins, see Fig. S8.

Among the factors influencing absorption wavelength, electrostatic interactions between the protein environment (residue charge) and PCB consistently contribute to lengthening the wavelengths by 40–60 nm (Table 1). Certain residues, such as the highly conserved basic residue forming a salt bridge with a propionic group of PCB (e.g., Lys956 in Anacy_2551g3; Fig. 6 *a*) and the highly conserved acidic residue forming an H-bond with a pyrrole N site of PCB (e.g., Asp92 in AnPixJg2; Fig. 6 *b*), influence the shortening and lengthening of absorption wavelengths, respectively. However, these effects are typically balanced by other electrostatic interactions with different residues (Fig. S8). As a result, differences among the PCB-binding proteins are generally limited to <20 nm (Table 1). A similar conclusion was reached in theoretical studies by Wiebeler et al., who demonstrated that the difference in the protein electrostatic interaction is not the primary factor for the significant variation in the absorption wavelength (10).

Notably, in Sb.phyB(PG)-PCB, the highly conserved acidic residue forming an H-bond with a pyrrole N site of PCB is present as Asp322. However, unlike in other PCB-binding proteins, in Sb.phyB(PG)-PCB, the side chain of Asp322 is not oriented toward the pyrrole N site due to the presence of Pro324; instead, it is the backbone carbonyl O site that interacts with the pyrrole N site of PCB. This exceptional orientation contributes to the lengthening, rather than the shortening, of the absorption wavelength in Sb.phyB(PG)-PCB (Fig. 6 *c*).

### Revisiting the lactim model hypothesized for the significantly lengthened absorption wavelength of Anacy_2551g3

The observation of a specifically red-shifted absorption wavelength in the Pfr state of the Anacy_2551g3 structure, even ∼50 nm longer than the next longest absorption wavelength in the Pr state of the RcaE structure, is noteworthy (Table 1). Based on the coplanarity of PCB in the QM/MM-optimized structure, its estimated absorption wavelength is 556 nm (Fig. 4), which is ∼170 nm shorter than the experimentally measured wavelength of 728 nm (Table 1).

**Figure 7:**
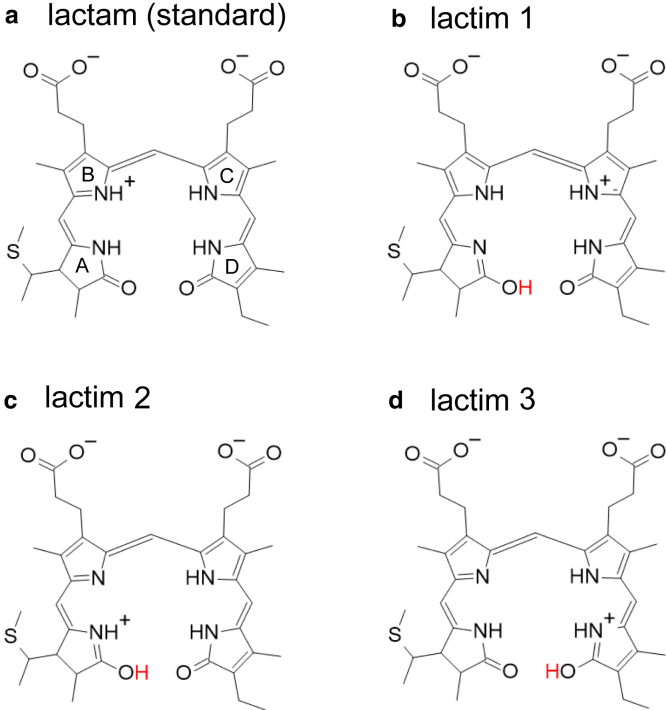
Lactim structures of PCB hypothesized by Bandara et al. (13). (*a*) Lactam. (*b*) Lactim 1 with deprotonated pyrrole N of ring A and protonated keto O of ring A. (*c*) Lactim 2 with deprotonated pyrrole N of ring B and protonated keto O of ring A. (*d*) Lactim 3 with deprotonated pyrrole N of ring B and protonated keto O of ring D. Protonated H atoms in lactim conformations are highlighted in red.

To explain this exceptional redshift, Bandara et al. proposed that PCB, typically assumed to be in the standard lactam conformation with all four N sites protonated, instead adopted a lactim conformation. In this lactim conformation, one of the two keto O sites is protonated due to deprotonation of one of the four N sites (13). Here, we investigated five possible lactim conformations, including all three speculated by Bandara et al. (Table 2; Fig. 7).

**Table 2:** QM/MM-optimized structures of Anacy_2551g3 with PCB in the lactim conformations

	Deprotonated N	Protonated O … acceptor	RMSD (Å)	Energy (kcal/mol)
Lactam (standard)	–	–	0.379	0
Lactim 1-1	N_ringA_	O_ringA_-H … O=C-Lys915	0.433	0.19
Lactim 1–2	N_ringA_	O_ringA_-H … O_ringD_	0.370	0.18
Lactim 2-1	N_ringB_	O_ringB_-H … O=C-Lys915	0.386	0.29
Lactim 2-2	N_ringB_	O_ringA_-H … O_ringD_	N/D	N/D
Lactim 3	N_ringB_	O_ringA_ … H-O_ringD_	0.342	0.12

See Fig. 7 for the molecular structures of the lactim conformations. RMSD, root-mean-square deviation of the QM/MM-optimized structure from the original crystal structure (Å); N/D, not determined due to its unstable conformation, resulting in the lactim 3 conformation during QM/MM/PCM calculations.

While among these conformations, the lactam conformation (i.e., the standard state with four protonated N sites) is found to be the most stable (Table 2), the lactim 3 conformation, with a protonated O site in ring D due to deprotonation of the ring-B N site, is only marginally less stable (Table 2). In particular, the QM/MM-optimized lactim 3 conformation exhibits the lowest root-mean-square deviation from the original Anacy_2551g3 crystal structure, even lower than the QM/MM-optimized lactam conformation (Table 2). However, it is essential to consider the resolution of the Anacy_2551g3 crystal structure, which was solved at 2.7 Å (13), significantly lower than that of all other crystal structures investigated in this study (ranging from 1.63 to 1.97 Å). At such a resolution, comparing only root-mean-square deviation values for assumed species fails to provide meaningful discrimination between lactam and lactim conformations.

Most importantly, the absorption energy for Anacy_2551g3, calculated for the lactim 3 conformation, is significantly deviated from the experimentally measured absorption energy (Fig. 2). The resulting absorption wavelength for the lactim 3 conformation is 608 nm, 120 nm blue shifted compared to the experimentally measured absorption wavelength of 728 nm (Table 1). In contrast, the absorption wavelength calculated for the standard lactam PCB conformation, as considered for all the other crystal structures of the PCB-binding proteins, is consistent with the experimentally measured absorption wavelength (Table 1; Fig. 2). These results suggest that the significantly red-shifted absorption wavelength of Anacy_2551g3 can unambiguously be explained as PCB in the standard lactam conformation without invoking the lactim hypothesis.

The significantly lengthened absorption wavelength of Anacy_2551g3 can be understood through the following molecular mechanism. Unlike in other investigated PCB-binding proteins (Fig. S5), in Anacy_2551g3, rings A and D are sufficiently close (N_ringA_ … N_ringD_ = 4.0 Å) to facilitate overlap of molecular orbitals between the two rings (Fig. 3 *c*). The HOMO exhibits antibonding character at the ring-A and -D moieties, destabilizing the HOMO. Typically, an increase in the HOMO energy level leads to a corresponding increase in the LUMO energy level (Fig. S9). However, for Anacy_2551g3, the LUMO exhibits bonding character at the ring-A and -D moieties, thereby stabilizing the LUMO (Fig. 3 *c*). Consequently, the overlap leads to a decrease in the HOMO-LUMO energy gap (Fig. S9), thereby lengthening the absorption wavelength. Hence, it seems most likely that the need for Bandra et al. (13) to postulate the unstable lactim species to rationalize the significantly red-shifted absorption wavelength of Anacy_2551g3 arises from the oversight of molecular orbital overlap between rings A and D.

It is important to note that deprotonation of the ring-N site itself results in significant blueshift regardless of the keto protonation (Fig. 2). Although it is true that the lactim 3 conformation (with a protonated O site in ring D due to deprotonation of the ring-B N site) exhibits a slight redshift compared to the standard lactam conformation with the deprotonated ring-B N site, this shift is too weak to account for the significantly red-shifted absorption wavelength of Anacy_2551g3 (Fig. 2).

Considering that in PCB-binding proteins, the absorption wavelength can be practically estimated from the PCB conformation, except for Anacy_2551g3 (Fig. 4), and assuming that the difference in absorption wavelengths between the estimated value of 556 nm (2.2 eV) derived from the coplanarity of PCB (Fig. 4) and the experimentally measured value of 728 nm (1.7 eV) (Table 1) originates from the molecular orbital overlap, the overlap of molecular orbitals would contribute to a decrease in the HOMO-LUMO energy gap of ∼0.5 eV in Anacy_2551g3. Therefore, the overlap of the molecular orbitals between rings A and D, rather than the protonation of the keto group (i.e., lactim formation), serves as the origin of the significantly red-shifted absorption wavelength of Anacy_2551g3. Indeed, RcaE lacks this overlap, resulting in a ∼50 nm shorter wavelength compared to Anacy_2551g3 (Table 1), despite a similar ring-like PCB shape (Figs. 3 *d* and S7).

## Conclusions

The present study provides a systematic approach for reproducing the absorption wavelengths of PCB-binding proteins using the original atomic coordinates of the crystal structures and calculating either the excitation energy (Table 1; Fig. 2) or the HOMO-LUMO energy gap (Fig. S4). Key residues, such as the highly conserved basic residue forming a salt bridge with a propionic group of PCB and the highly conserved acidic residue forming an H-bond with a pyrrole N site of PCB, influence the shortening and lengthening of absorption wavelengths, respectively (Figs. 6 and S8). Nonetheless, these effects are often offset by other electrostatic interactions with different residues, resulting in differences of <20 nm among the PCB-binding proteins (Table 1).

In contrast, differences in absorption wavelengths among PCB-binding proteins primarily originate from the PCB conformation (Table 1), as indicated by the high correlation observed between the absorption wavelengths of isolated PCB molecules and those of PCB-binding proteins (Fig. S6). Notably, the coplanarity of PCB can reliably estimate absorption wavelengths of PCB-binding proteins except for Anacy_2551g3 (Fig. 4). The PCB conformation also plays a crucial role in determining the loss of solvation for PCB in the protein environment (Fig. 5), which shortens the absorption wavelength, differentiating absorption wavelengths by ∼50 nm among PCB-binding proteins (Table 1).

Anacy_2551g3 exhibits a significantly lengthened absorption wavelength of 728 nm. While lactim formation slightly extends the absorption wavelength, it does not sufficiently explain the observed lengthened absorption wavelength for Anacy_2551g3 (Fig. 2). Instead, the pronounced overlap of molecular orbitals between rings A and D, particularly notable in Anacy_2551g3 (Fig. 3 *c*), contributes to a decrease in the HOMO-LUMO energy gap (Fig. S9), thereby lengthening the absorption wavelength.

Overall, the methodology presented here enables the accurate estimation of absorption wavelengths of PCB-binding proteins based on the original crystal structures, providing a common framework for understanding the molecular mechanisms underlying color tuning. This approach has already successfully reproduced absorption wavelengths in microbial rhodopsins (16,17) and photoreceptors such as photoactive yellow proteins (36), demonstrating its broad applicability. It holds significant potential for advancing the study of various pigment-containing proteins, including photosynthesis reaction centers and light-harvesting complexes.

## Author contributions

T.N. and H.I. designed the research; T.N. performed computations; T.N., K.S., and H.I. analyzed the data; and H.I. wrote the paper.

## Supporting material

Document S1. Tables S1–S5 and Figures S1–S9

Data S1. PCB coordinates

Document S2. Article plus supporting material
