# Supplementary material for: Molecular origins of absorption wavelength variation among phycocyanobilin-binding proteins
Source: Biophys J. 2024 Aug 8;123(19):3375–85. doi: 10.1016/j.bpj.2024.08.001 (PMC11480761; doi:10.1016/j.bpj.2024.08.001)
Supplement: Document S1. Tables S1–S5 and Figures S1–S9 [file mmc1.pdf]

Biophysical Journal, Volume *123*

Supplemental information

Molecular origins of absorption wavelength variation among phycocyanobilin-binding proteins

Tomoyasu Noji, Keisuke Saito, and Hiroshi Ishikita

Supporting Material

Molecular origins of absorption wavelength variation among phycocyanobilin-binding proteins

Tomoyasu Noji [1,2], Keisuke Saito [1,2], and Hiroshi Ishikita [1,2]*

1) Department of Applied Chemistry, The University of Tokyo, 7-3-1 Hongo, Bunkyo-ku, Tokyo 113-8654, Japan

2) Research Center for Advanced Science and Technology, The University of Tokyo, 4-6-1 Komaba, Meguro-ku, Tokyo 153-8904, Japan

CORRESPONDING AUTHOR: Ishikita, Research Center for Advanced Science and Technology, The University of Tokyo, 4-6-1 Komaba, Meguro-ku, Tokyo 153-8904, Japan, Tel. +81-3-5452-5056, Fax. +81-3-5452-5083, **E-mail:** hiro@appchem.t.u-tokyo.ac.jp

Table S1. Atomic partial charges of PCB.

tetraphyrrole	atomic charge
CHA	-0.08
HHA	0.16
NA	-0.35
HNA	0.32
C1A	0.04
C2A	0.05
C3A	0.10
C4A	0.13
CMA	-0.38
HMA1	0.13
HMA2	0.13
HMA3	0.13
CAA	-0.24
HAA1	0.14
HAA2	0.14
CHB	-0.28
HHB	0.15
NB	-0.52
HN	0.33
C1B	0.18
C2B	0.14
C3B	-0.23
C4B	0.60
CMB	-0.42
HMB1	0.14
HMB2	0.14
HMB3	0.14
CAB	0.12
HAB1	0.03
HAB2	0.03
CBB	-0.24
HBB1	0.07
HBB2	0.07
HBB3	0.07
OB	-0.45
NC	-0.35

HNC	0.31
C1C	0.43
C2C	0.10
H2C	0.05
C3C	0.02
H3C	0.05
C4C	0.14
CMC	-0.25
HMC1	0.08
HMC2	0.08
HMC3	0.08
CAC	-0.05
HAC1	0.09
CBC	-0.20
HBC1	0.07
HBC2	0.07
HBC3	0.07
OC	-0.44
CHD	-0.39
HHD	0.18
ND	-0.20
HND	0.25
C1D	0.18
C2D	0.01
C3D	0.09
C4D	-0.05
CMD	-0.24
HMD1	0.10
HMD2	0.10
HMD3	0.10
CAD	-0.21
HAD1	0.12
HAD2	0.12
**total**	1.00
**cysteine**	
1CB	-0.1
HB1	0.09
HB2	0.09
1SG	-0.08
**total**	0

**ring-B propionic group**	deprotonated	protonated
CBD	-0.28	-0.21
HBD1	0.09	0.09
HBD2	0.09	0.09
CGD	0.62	0.75
O1D	-0.76	-0.36
O2D	-0.76	-0.36
**total**	-1	0
**ring-C propionic group**	deprotonated	protonated
CBA	-0.28	-0.21
HBA1	0.09	0.09
HBA2	0.09	0.09
CGA	0.62	0.75
O1A	-0.76	-0.36
O2A	-0.76	-0.36
**total**	-1	0

Table S2. Titratable residues showing discrepancies in calculated protonation states between the present approach and PROPKA 3. For histidine, residues calculated to be doubly protonated in at least one of the two approaches are listed. Protonation probabilities in PROPKA 3 were calculated, using the Henderson-Hasselbalch equation with the resulting pK_a at pH 7. Residues not listed are in their standard protonation states, i.e., protonated basic and deprotonated acidic residues.

		**H^+ probability**		
		**present approach**	**PROPKA 3 (pK_a)**	**from PCB (Å)**
Anacy_2551g3	His863	0.75	0.13 (6.18)	>20
	His1021	0.61	0.29 (6.62)	>20
AnPixJg2	His119	0.99	0.12 (6.12)	2.9
PPHK	His52	0.64	0.14 (6.22)	>25
	His137	0.86	0.17 (6.10)	>25
	His242	0.55	0.56 (7.10)	2.58
RcaE	Glu143	0.00	0.93 (8.11)	2.7
	His239	0.95	0.64 (7.25)	>10
	His257	0.68	0.29 (6.61)	>10
Sb.phyB(PG)-PCB	His208	0.71	0.27 (6.56)	>25
	His294	0.51	0.22 (6.44)	>10
	His298	0.61	0.59 (7.15)	>10
Slr1393g3 (Pg)	His529	0.96	0.07 (5.85)	2.7

Table S3. QM regions involving the H-bond network.

	**chromophore**		**H-bond network**			**water**
	PCB	cysteine	side-chain	backbone	both	
Anacy_2551g3	PCB	Cys943	Tyr924, Arg930, Lys956, His989, Tyr947	Lys915	Glu914	
AnPixJg2	PCB	Cys122	Trp90, Asp92, Arg102, Tyr103, His123, Tyr153	Phe120	His119	301, 302, 392
PPHK	PCB	Cys241	Asp211, Arg218, Gln228, His242, Asn254			
RcaE	PCB	Cys248	Glu217, Tyr227, Lys261, His285			539, 544, 548, 556, 580
Sb.phyB(PG)-PCB	PCB	Cys372	Arg337, Arg367, His419	Asp322, Ile323, His370	Pro369	613, 623, 639, 669, 681, 696
Slr1393g3 (Pg)	PCB	Cys528	Asp498, Arg508, His529			811, 823, 832, 848
Slr1393g3 (Pr)	PCB	Cys528	Trp496, Asp498, Thr526, His529, Thr543, Tyr559		Gly507, Arg508	805, 820, 823

Table S4. Protonation states of propionic groups at the pyrrole rings B and C of PCB ([H^+]). 0 H^+ corresponds to the fully deprotonated state, whereas 1 H^+ corresponds to the fully protonated state.

	**ring B**	**ring C**
Anacy_2551g3	0.00	0.00
AnPixJg2	0.00	0.00
PPHK	0.03	0.00
RcaE	0.00	0.00
Sb.phyB(PG)-PCB	0.00	0.00
Slr1393g3 (Pg)	0.00	0.02
Slr1393g3 (Pr)	0.00	0.00

Table S5. Experimentally measured (expl.) and calculated (calc.; E_{TDDFT}) absorption energies of PCB in the presence (protein) /absence (water) of the PCB-binding protein environments (eV).

	**state**	**expl.**	**calc.**	
			**protein**	**water**
Anacy_2551g3	Pfr	1.704	2.056	2.147
AnPixJg2	Pr	1.915	2.258	2.329
PPHK	Pg	2.208	2.455	2.426
RcaE	Pr	1.874	2.175	2.255
Sb.phyB(PG)-PCB	Pr	1.942	2.250	2.257
Slr1393g3	Pg	2.315	2.556	2.562
	Pr	1.912	2.220	2.315

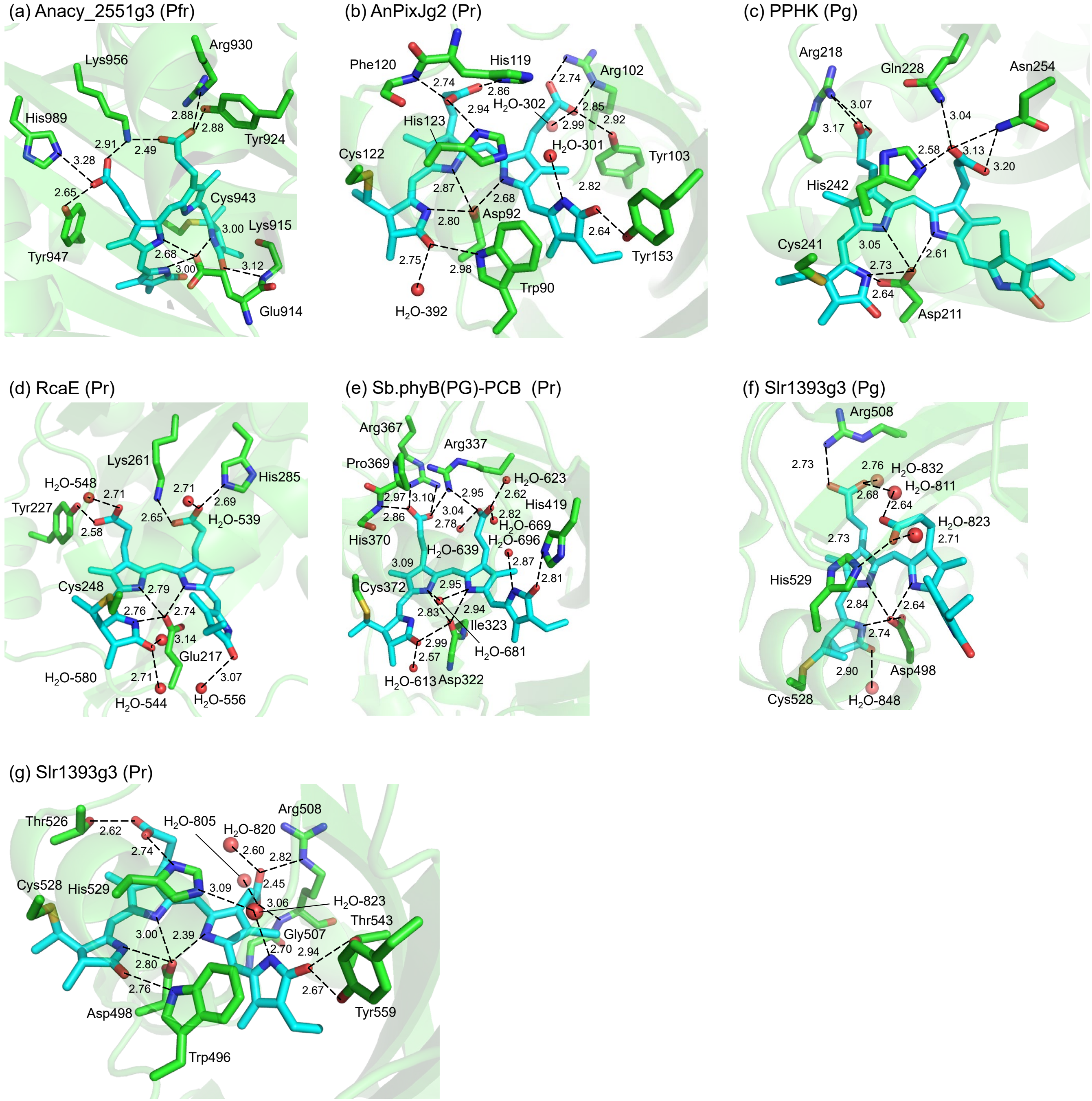

Figure S1. PCB chromophores in the crystal structures of the PCB-binding proteins. PCB and H-bond network groups, considered quantumchemically in QM/MM/PCM calculations (i.e., QM region), are shown explicitly. Dotted lines indicate H-bonds.

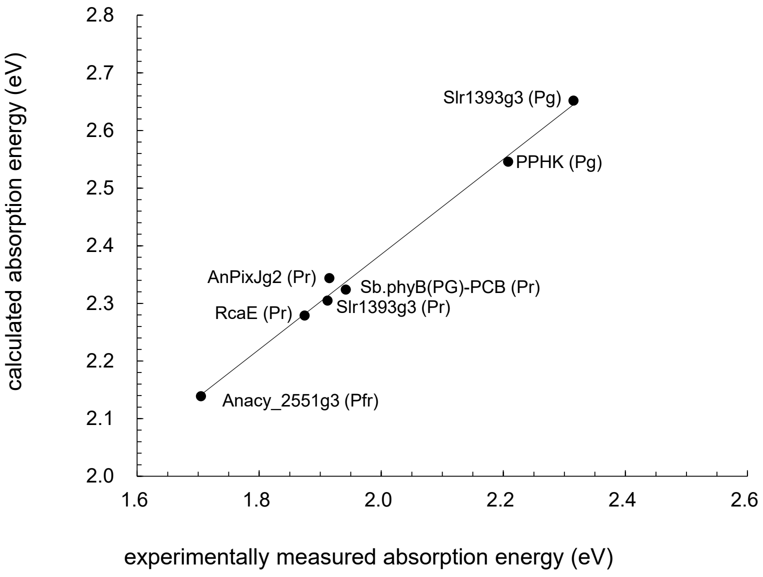

Figure S2. Comparison of calculated absorption energies and experimentally measured absorption energies for PCB-binding proteins. Absorption energies calculated considering the residues involved in the H-bond network of the PCB moiety quantumchemically (i.e., QM region). Geometry optimization was perfumed using the CAM-B3LYP functional and LACVP* basis sets with a range-separation parameter μ of 0.33, α of 0.19, and β of 0.46.

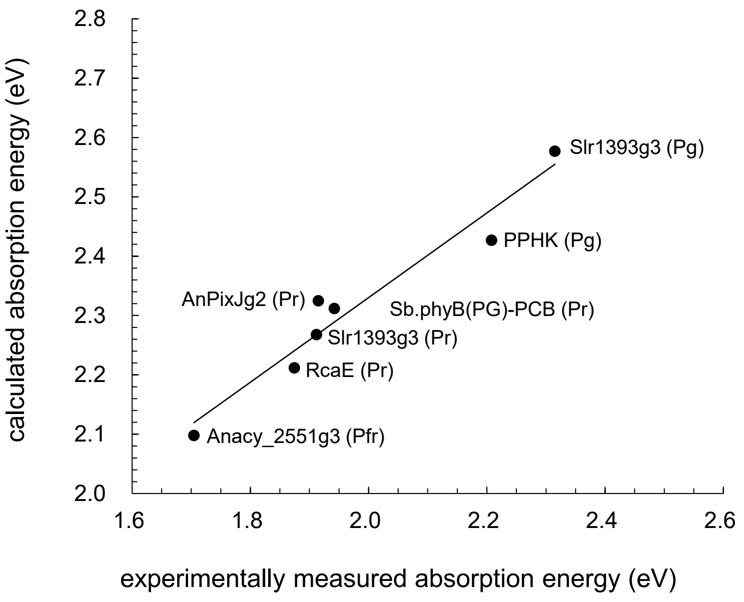

Figure S3. Absorption energies calculated considering the residues involved in the H-bond network of the PCB moiety quantumchemically (i.e., QM region) when calculated without PCM (coefficient of determination $R^2 = 0.94$).

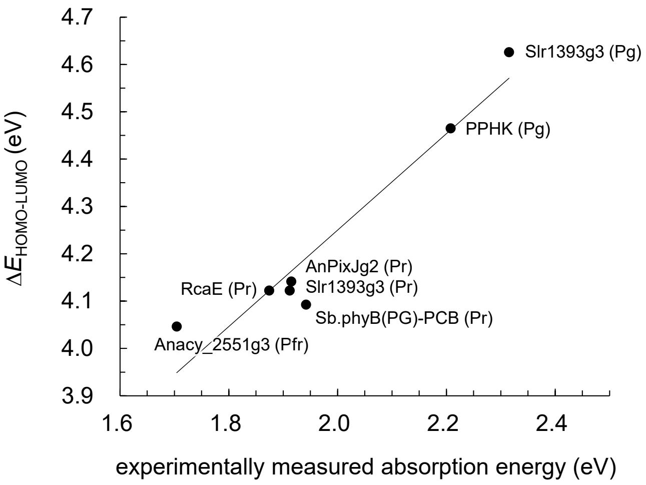

Figure S4. Comparison of calculated HOMO-LUMO energy gap ($\Delta E_{\mathrm{HOMO\text{-}LUMO}}$) and experimentally measured absorption energies for PCB-binding proteins. This correlation is best described by the following equation (coefficient of determination $R^2 = 0.92$): E_{expl} (eV) = 0.981 $\Delta E_{\mathrm{HOMO\text{-}LUMO}} - 2.170$ (eq. S1).

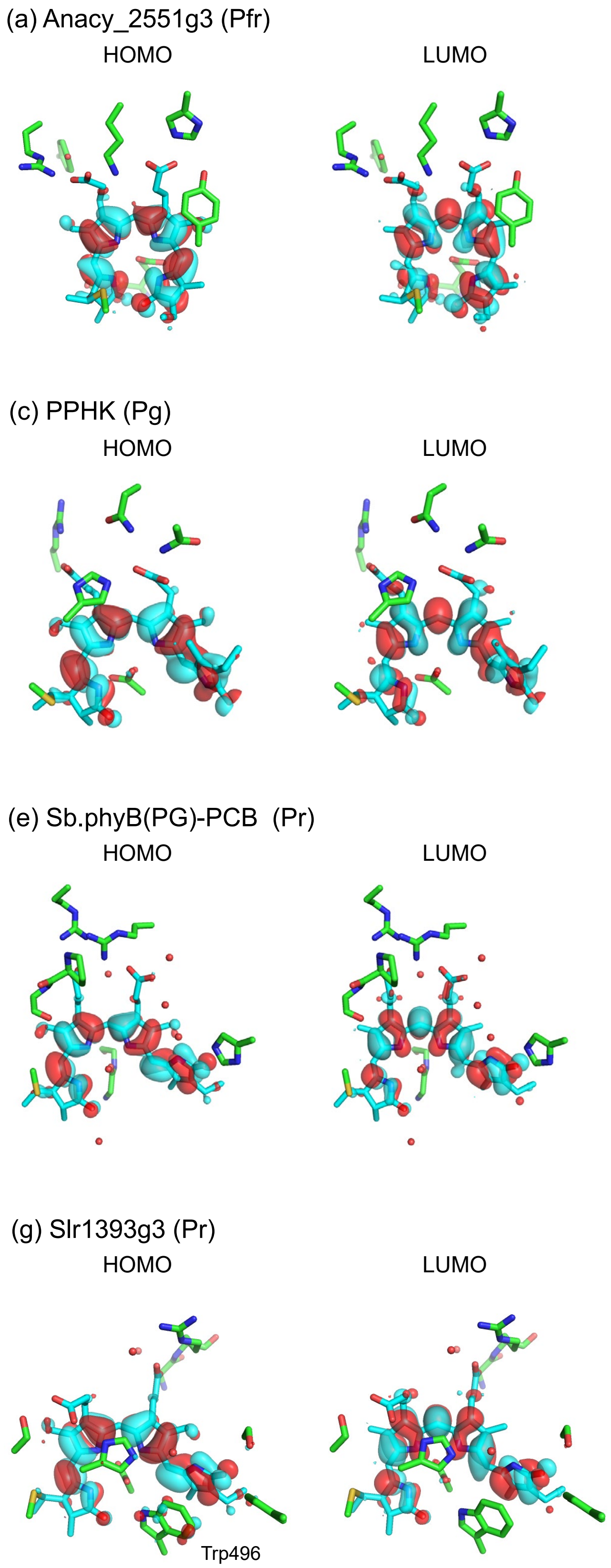

Figure S5. Distributions of the HOMO and LUMO over the PCB chromophores in the presence of the protein environment. All H-bond network groups are involved in the QM region.

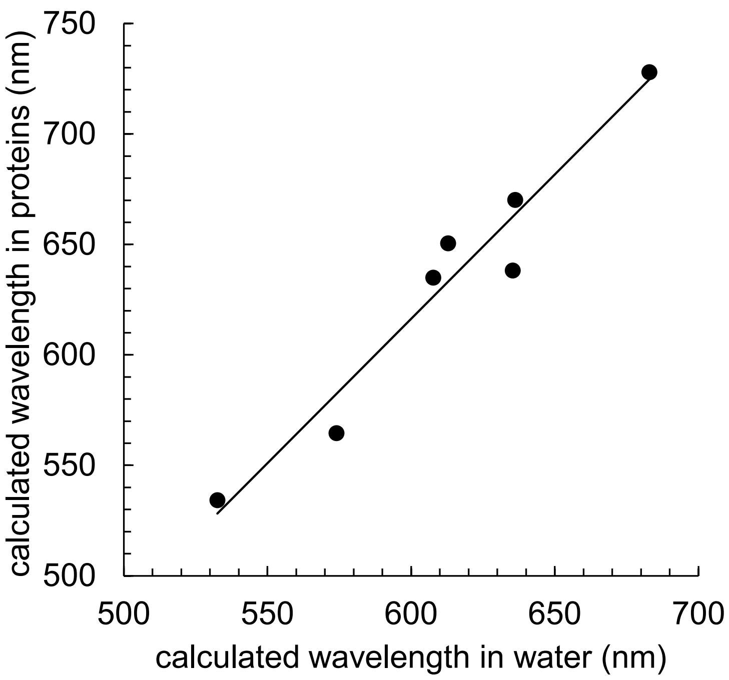

Figure S6. Comparison of calculated absorption wavelengths (λ) in PCB-binding proteins and water listed in Table 1. This correlation is best described by the following equation (coefficient of determination R^2 = 0.95): λ (nm) = 1.306 λ_{water} – 167.5 (eq. S2).

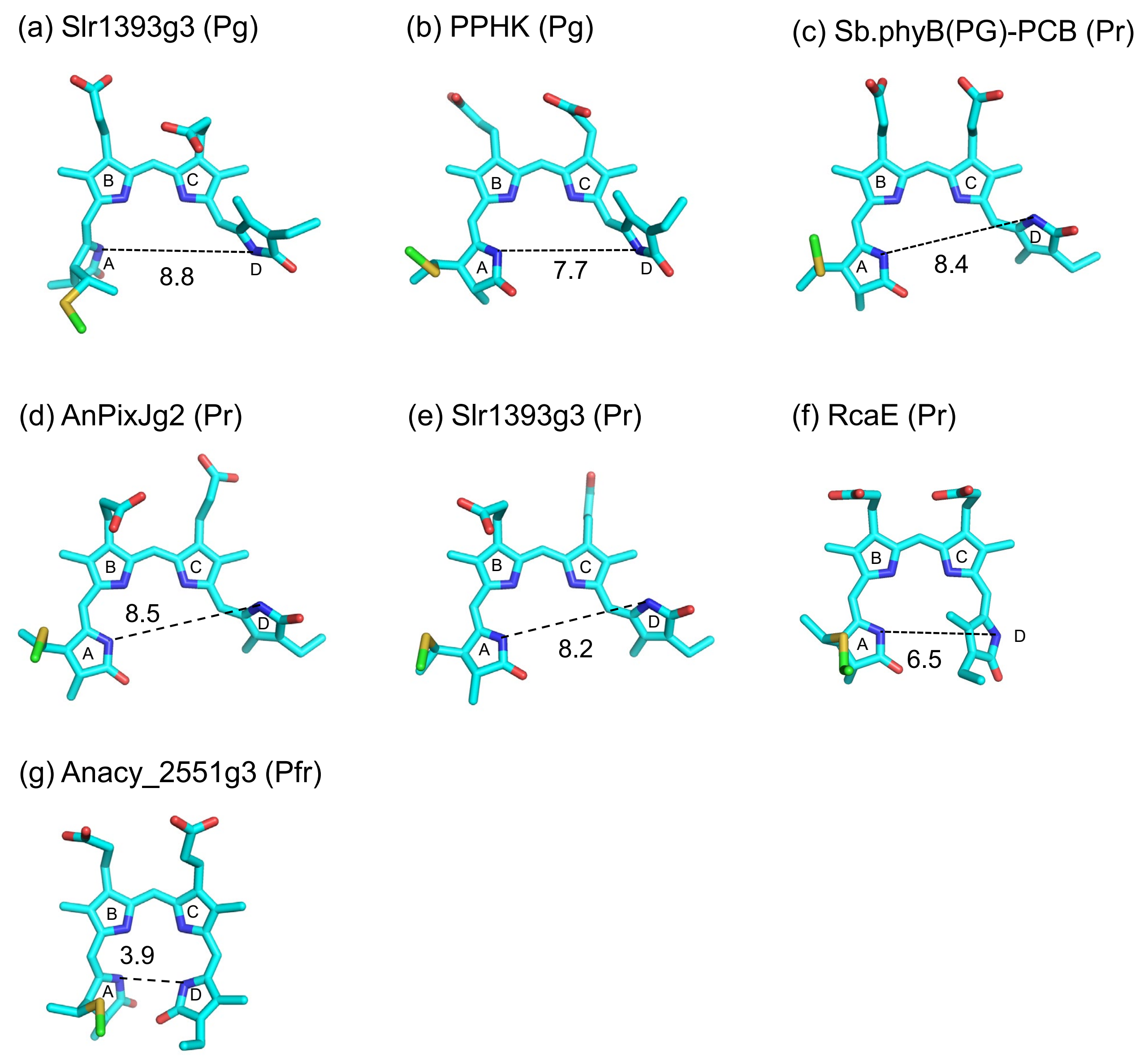

Figure S7. PCB conformations in the QM/MM-optimized structures of the PCB-binding proteins. The QM region includes PCB and H-bond network groups. Dotted lines indicate distances between N sites in rings A and D.

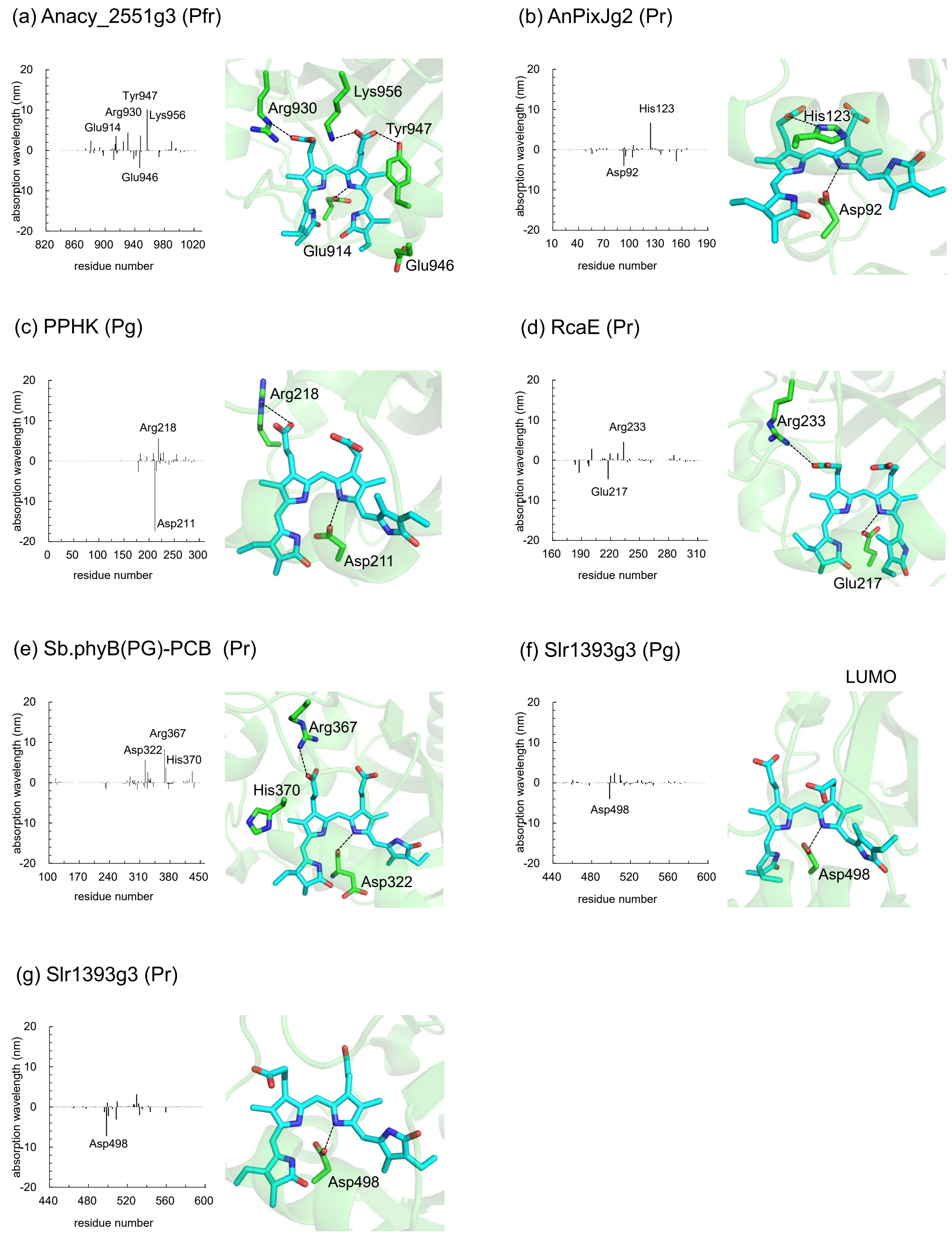

Figure S8. Residues that electrostatically affect the absorption wavelengths in PCB-binding proteins.

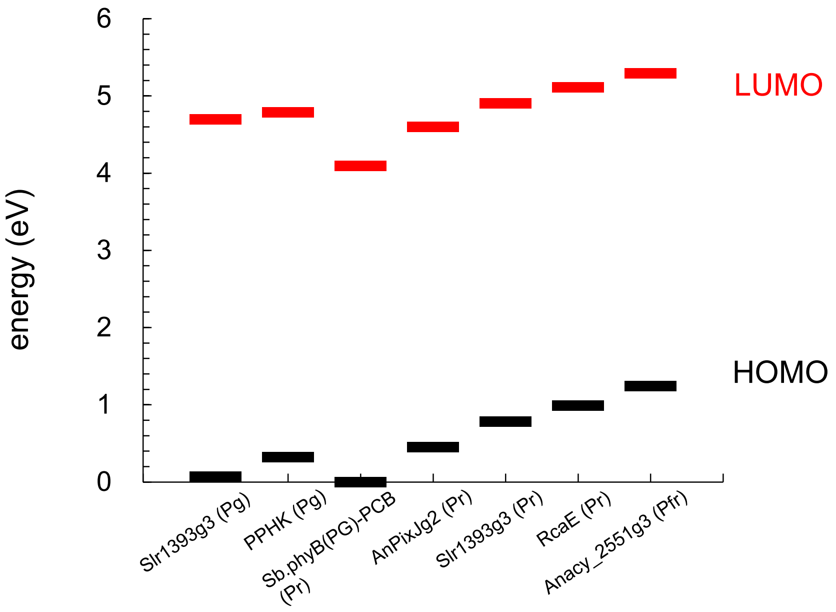

Figure S9. Energy levels of HOMO and LUMO in the presence of the protein environment. All H-bond network groups are involved in the QM region.
